# Causes, consequences and biomarkers of stress in swine: an update

**DOI:** 10.1186/s12917-016-0791-8

**Published:** 2016-08-19

**Authors:** Silvia Martínez-Miró, Fernando Tecles, Marina Ramón, Damián Escribano, Fuensanta Hernández, Josefa Madrid, Juan Orengo, Silvia Martínez-Subiela, Xavier Manteca, José Joaquín Cerón

**Affiliations:** 1 Department of Animal Production, Campus of Excellence Mare Nostrum, Universidad de Murcia, 30100 Murcia, Spain; 2 Department of Animal Medicine and Surgery, Campus of Excellence Mare Nostrum, Universidad de Murcia, 30100 Murcia, Spain; 3 Department of Animal and Food Science, Universitat Autònoma de Barcelona, 08193 Bellaterra, Barcelona, Spain

**Keywords:** Stress, Pigs, Biomarkers

## Abstract

**Background:**

In recent decades there has been a growing concern about animal stress on intensive pig farms due to the undesirable consequences that stress produces in the normal physiology of pigs and its effects on their welfare and general productive performance. This review analyses the most important types of stress (social, environmental, metabolic, immunological and due to human handling), and their biological consequences for pigs. The physio-pathological changes associated with stress are described, as well as the negative effects of stress on pig production. In addition an update of the different biomarkers used for the evaluation of stress is provided. These biomarkers can be classified into four groups according to the physiological system or axis evaluated: sympathetic nervous system, hypothalamic-pituitary-adrenal axis, hypothalamic-pituitary-gonadal axis and immune system.

**Conclusions:**

Stress it is a process with multifactorial causes and produces an organic response that generates negative effects on animal health and production. Ideally, a panel of various biomarkers should be used to assess and evaluate the stress resulting from diverse causes and the different physiological systems involved in the stress response. We hope that this review will increase the understanding of the stress process, contribute to a better control and reduction of potential stressful stimuli in pigs and, finally, encourage future studies and developments to better monitor, detect and manage stress on pig farms.

## Background

The worldwide increase in the demand for animal products in recent decades has led to the use of intensive systems that have been demonstrated to produce stress in animals [1]. Since Hans Selye first introduced the concept of stress as “the non-specific response of the body to any demand for change” [2], the concept has received several definitions, so that nowadays its meaning can vary depending of the biological field in which it is used. According to Fink [3], stress in behavioral sciences is regarded as the perception of threat, with resulting anxiety, discomfort, emotional tension, and difficulty in adjustment. In terms of pure neuroendocrinology, stress is any stimulus that provokes release of the adrenocorticotropic hormone (ACTH) and adrenal glucocorticoids; and for the sociologists, it is any social disequilibrium that produces disturbances in the social structure within which a population lives [3]. Despite this variability in meaning, one of the most accepted definitions of stress is “the biological response elicited when an individual perceives a threat to its homeostasis” [4] which can apply to both humans and animals. In this review we will study the main types of stress that can affect pigs in intensive farming conditions, its consequences and the main biomarkers that can be used to detect whether the pigs are enduring stressful conditions.

Various classifications of stress have been described in the literature. From a practical point of view, it is of interest to classify the stress according to its duration and also to its causes. Regarding the duration of the stress, it can be acute (short in duration; lasting minutes or various days) or chronic (lasting weeks, months or even years). In addition, depending of its cause, the stress can be classified as social, environmental, metabolic, immunological or due to human practices and manipulation of animals.

## Main causes of stress

In the following lines we will study the main causes of stress, as well the situations in the current intensive production systems where the pigs can be more exposed to these causes. It is important to point out that different external factors can produce a similar stress response, but on the other hand, the same stressful stimulus can produce a different response in the animal depending on its age, genetics, production system or previous exposure to the stimulus.

### Social stress

Pigs are regrouped with unfamiliar conspecifics at different times of their productive cycle such as during gestation or after weaning, during the fattening period or before transport to slaughter. In these situations, pigs may fight in order to establish a new dominance hierarchy and this generates stress [5]. Such social stress can be acute, immediately following regrouping, or chronic, when the animals are socially subordinate or isolated [6], or as a result of repeated social regrouping [7].

Social stress can vary depending of the group size, space available and gender and genetic of the pig. Larger groups seem to have less social stress than smaller groups, reflecting a lower probability of monopolizing resources as the group size increases [8]. Intensive housing involves a reduction in the space per animal, which causes stress because of restricted movements and freedom to feed themselves [9]. It has been demonstrated that the frequency of social interactions and aggressive behaviour increases as the space allowance decreases in group-housed sows [10, 11] and the growth rate decreases linearly as the space allowance per pig decreases [12]. Response to social stress is higher in males than in females [13]. Aggressive behavior after regrouping varies between individuals and to some extent such individual differences have a genetic basis. As a result, some lines of pigs seem to fight less than other when unacquainted individuals are mixed [14].

### Environmental stress

Intensive pig farming requires control of the temperature, humidity, light, concentration of dust and gases, ammonia levels and sound intensity [15–20]. For example, ambient temperature should be as close as possible to the thermal neutrality for the age of the pig being housed, (i.e. for prenursery pigs between 28 and 32 °C, for nursery pigs between 22 and 28 °C (depending on live weight, with lower live weight requiring higher temperature), for growing animals close to 20 °C, for finishing pigs between 16 and 18 °C, for pregnant sows between 15 and 18 °C, and for lactating sows and boars close to 16 °C) [15]. However, sometimes optimal environmental conditions cannot be maintained in farms producing stress in animals. For example, in areas where there are extreme hot or cold seasons, in farms located in areas of high noise or in the case of equipment failure. It is important to bear in mind that the effects of the thermal environment on pigs do not depend solely on ambient temperature, but on the so-called effective temperature, which depends on ambient temperature, ventilation and flooring, among other factors.

It should also be pointed out that the barren environments of modern production systems have a negative effect on animal welfare due to the lack of bedding material and/or slatted pen floors. They create a poor environment where pigs cannot develop their natural behaviour [21]. Inability to perform highly motivated behaviors may lead to a stress response. This is the case, for example, when sows are prevented from showing nest-building behavior before farrowing [22].

### Metabolic stress

This stress results from food and/or water restriction or deprivation [23–25]. Although most studies in the literature induce metabolic stress by means of experimental models, metabolic stress can also appear in intensive farming conditions when pregnant sows are subjected to restrict feeding, which has been shown to result in chronic hunger [26]. Likewise, in the process of mixing pregnant sows, it is usual for the more submissive animals to have less or no access to food [27].

In any case, the negative effects of food deprivation will depend on the length of the fasting period. When an animal is food-deprived, its glucose levels in blood drop and enters in a catabolic state that requires the use and production of alternative fuels such as non-esterified fatty acids (NEFA), beta-hydroxybutyrate and glycerol for energetic needs [28, 29]. These analytes could be used as indicators of metabolic stress [29].

### Immunological stress

Immunological stress is produced when an animal is challenged by infectious agents [30], which may occur due to the presence of disease or after vaccination. Stress can be considered as a possible cause but also a consequence of infectious disseases. A situation of stress produces changes in numbers and proportions of blood leukocytes [31], mitogen-induced cell proliferation, natural killer cell cytotoxicity and circulating inflammatory factors [32], which can result in increased susceptibility to any infectious disease [33]. And in the case of an existing disease, the stress associated may predispose pigs to the emergence of a different disease that complicates the process [13, 34].

### Stress by animal handling

On commercial farms, pigs are subjected to handling practices which can cause acute stress. Some of these practices such as snaring or vena cava blood sampling produce a high stress, whereas others such as tattoing or electric shocks induced a more moderate stress in a previous study [35]. Similarly, castration, a common operation on some farms, has been shown to cause stress when carried out without anesthetics or analgesics [36].

### Situations in intensive production systems where pigs are more exposed to stressors

There are two particular situations in the productive system, namely weaning and transport, when animals are more exposed to the causes of stress described above, and which generate a high degree of stress in the animals. Weaning is a period full of challenges to the piglet such as abrupt separation from the sow, transportation to a new physical environment, a different food source, commingling with pigs from other litters, greater exposition to pathogens and dietary or environmental antigens and handling practices such as vaccination and drug administration [37].

Transport either to another holding or slaughterhouse also includes exposure to different stressful events such as departure from the usual room, truck loading and unloading, fasting, different temperature and humidity, noise, vibration of the vehicle or inappropriate stocking densities [16, 38].

Therefore, special care should be taken in weaning, providing high-quality diets to minimize the risk of diarrhoea and reinforce immune system. Highly digestible animal proteins and feed additives such as enzymes and dietary acids may be used to improve the digestibility of the diet. Another option would be to offer low-protein and amino acid-fortified diets to limit the amount of fermentable protein presented to the gut and the use of pre- and probiotics, n-3 long chain polyunsaturated fatty acids and n-6 linoleic acid. Moreover, special attention should be paid to environmental and sanitary conditions in the post weaning stage, and an all-in, all-out management system should be used to minimize disease exposure [37, 39].

In addition, there are published reports and official documents with guidelines to improve the welfare and production of pigs during transport [16, 40]. These guidelines include avoiding extreme temperatures and the overloading of trucks, minimizing the time the pigs are on the truck, using non-slip flooring on loading ramps and providing an adequate handling to the pigs by well-trained people [16, 40].

## Consequences of stress in pigs

### Biological response

The stressors mentioned above are perceived by pigs as a threat to their homeostasis, which trigger a variety of biological responses (behavioural, neuroendocrine and immunological).

#### Behavioural responses

Avoiding a threat, facing up to it or hiding from it can be described as normal behaviour, whereas stereotypes (a repetitive, invariant behaviour pattern with no obvious goal or function) are considered as abnormal behaviours that can appear after a stress [41]. In addition, frequent defecation can be an indicator of fear and stress, and excessive aggressive behaviour as well as tail and ear biting are considered abnormal behaviours [42, 43]. A behavioural ethogram to evaluate the external response of pigs to stress has been proposed by Ruis et al. [44], which covers exploring, defecation/urination, inactive (sleeping, lying, sitting and standing), ingestive (feeding and drinking), vocalizing and walking behaviours.

#### Sympathetic-adrenal-medullary axis response (SAM)

Catecholamines (epinephrine and norepinephrine) are released by the chromaffin cells of the adrenal medulla when the sympathetic nervous system is activated by a stress stimulus. An increase in heart rate and blood pressure is one of the consequences of this activation. Other changes, such as constriction of the intestine and skin vessels, dilatation of skeletal muscle vessels, dilatation of the pupils, or release of glucose and lipids from the liver are also observed. All these short-term changes prepare the animal to face the stressful stimulus or to escape from it [45].

#### Hypothalamic-pituitary-adrenal axis response (HPA)

Corticotropin-releasing factor is released by the hypothalamus once the stressful stimulus is detected. As a consequence of this, the adrenocorticotropic hormone (ACTH) is released by the anterior pituitary gland. Finally, glucocorticoids are produced and released by the adrenal cortex to increase the catabolism of tissues rich in protein and fat to produce glucose [45].

#### Hypothalamic-pituitary-gonadal axis response (HPG)

Stress is generally accompanied by both an increase in the HPA activity and a decrease in HPG [46]. Glucocorticoids inhibit the release of luteizing hormone (LH) from the pituitary and estradiol and progesterone secretion by the ovary [47], as well testosterone from the testes [48], decreasing blood concentrations of these hormones. Prolonged or chronic stress usually results in inhibition of reproduction, while the effect of transient or acute stress in certain cases may be stimulatory [49].

#### Immune system response

This response depends on the mediator released in the process of stress: catecholamines or glucocorticoids [50]. Catecholamines, which are usually produced at the beginning of stress or during a short-term stressful stimulus, produce an increase in leukocytes (mainly granulocytes and lymphocytes), which are released into the systemic circulation. On the other hand, cortisol which predominates when stressful stimuli are prolonged, mainly decreases the number of lymphocytes in blood. Glucocorticoids can also suppress the production of cytokines and immunoglobulins [50–52].

### Consequences for pig production

Pigs in intensive systems have to cope with long-term and intense short-term stressful stimuli that affect their welfare. High levels of stress and poor welfare have negative effects on five main factors related with pig production: pig performance, reproduction, behaviour, immunity and meat quality [53]. The magnitude of these effects will vary depending on stress duration and intensity, and on the early experience, age and genetics of the animal [4]. Some representative examples of these negative effects are provided below.

Any stressful condition can produce a decrease in performance parameters of pigs such as feed intake, daily gain or body weight (Table 1). For example, pigs housed at 32.2 °C had a 32.3 % decrease in average daily feed intake, 39.3 % lower average daily gain, 9.8 % lower final body weight and a 16.3 % lower gain:feed ratio compared with pigs housed at 23.9 °C [17]. Indeed, feed intake can decrease by up to 47 % in pigs housed at temperatures above the thermoneutral zone [19]. Similarly, Hyung et al. [54] found that growth rates were reduced by 15.7 and 7.1 % as a result of crowding and mixing, respectively, and showed a decrease in daily gain and gain:feed ratio of about 15 and 10 %, respectively, when the space available per pig was reduced from 0.56 to 0.25 m^2^/pig. Similarly, Lee et al. [55] showed that weaner pigs housed in a clean environment consumed about 8 % more feed and grew faster (about 10 %) than those housed in a dirty environment, and Hicks et al. [56] described that shipping by 4 h in weaner pigs produced a loss of 2.9 % body weight.

**Table 1:** Summary of stressful stimuli that produce a decreased in pig performance

Parameter affected	Stressful stimuli	Decrease, %	Reference
ADFI	Heat stress	32.3	White et al. [17]
		47.0	Pearce et al. [19]
	Dirty environment	8.0	Lee et al. [55]
ADG	Heat stress	39.3	White et al. [17]
	Crowding	15.7	Hyung et al. [54]
	Mixing	7.1	Hyung et al. [54]
	Decreasing space availability	15.0	Hyung et al. [54]
	Dirty environment	10.0	Lee et al. [55]
BW	Heat stress	9.8	White et al. [17]
	Shipping (4 h)	2.9	Hicks et al. [56]
G:F	Heat stress	16.3	White et al. [17]
	Decreasing space availability	10.0	Hyung et al. [54]

*ADFI* average daily feed intake, *ADG* average daily gain, *BW* body weight, *G:F* Gain:Feed ratio

A negative effect on the reproductive system has been described in boars in situations of stress, with a reduction in both ejaculate volume and semen quality, while gilts and sows may show fewer born piglets per litter and reduced rebreeding rate, as well as irregular rebreeding, higher weaning-to-oestrus interval [49], leading to a fall in farm production parameters.

In stressful situations there may be a reduction in the normal function of the immune system [31, 34], which may even suppress the response after vaccination [13]. This will result in an increase in the presence of diseases [57], with a subsequent increase in costs and decrease in production.

In addition, stress affects meat quality and an increase in incidence of pale, soft and exudative (PSE) and dark, firm and dry (DFD) meats, and reduced meat quality was found when stressful handling systems were used [16].

## Biomarkers of stress studied in pigs

There is no a “gold standard” procedure to determine with accuracy the degree of animal welfare and the level of stress of an animal. Methodologies frequently used to quantify stress in animals include: (1) direct behavioural observations using behaviour scoring systems [44, 53, 58] or automated behaviour recognition video analysis [59, 60], and (2) biomarkers that can reflect the pathophysiological responses to stress [61–63]. There is a tendency to evaluate a panel of various biomarkers representing the different body systems involved: SAM, HPA axis, HPG axis and immune system.

### Sympathetic Nervous System: alpha-amylase and chromogranin A

#### Alpha-amylase

This enzyme is used in humans as a stress biomarker because it increases after both physical and psychological stress [64]. It is measured in saliva and its activity is correlated with plasma catecholamine concentrations, being a marker of SAM activation [64–66]. In pigs alpha-amylase activity increases after 1 min of immobilization with nasal snares, however, high inter-individual variability in the responses was observed and some pigs did not present evident changes after this stressful stimulus [67]. Further studies would be necessary to clarify the role of this enzyme in porcine stress.

#### Chromogranin A

Chromogranin A (CgA) is an acidic soluble protein belonging to the granins family [68]. It was initially detected in chromaffin granules of the adrenal medulla, [69]. Later, CgA was found in secretory vesicles of endocrine, neuroendocrine and neuronal cells [70, 71]. During stress CgA is secreted into the blood by the adrenal medulla and anterior pituitary gland, with a smaller amount also released from sympathetic nerve endings [68]. In addition, CgA has been detected in salivary glands of humans and various animal species [72–74]. The release of CgA in saliva is mediated by the secretion of catecholamines [75], and has been related to the activation of SAM [76]. In humans, the measurement of CgA in saliva has been used as a reliable marker of SAM activation as an alternative to adrenaline and noradrenaline [77], which have high variability in results and low stability in saliva [78–80].

In pig, CgA has been used as a marker of SAM activation in different situations such as immobilization with a nasal snare [80], after refeeding following a period of food deprivation [25] and after isolation or regrouping [63]. CgA is not affected by age, gender or circadian rhythms [81]. This could be considered as an advantage compared with other stress biomarkers such as cortisol [82, 83], or Ig A [84] which are both influenced by these factors.

### Hypothalamic-Pituitary-Adrenal Axis: Glucocorticoids

Cortisol is one of the most widely used biomarkers to detect stress in pigs, and is the main glucocorticoid in this species [85]. Its increase is related with HPA axis activation [86–88].

Despite its wide use, cortisol is influenced by factors such as circadian rhythm and genetics that could limit its use as a stress biomarker [89, 90]. Cortisol concentrations follow a circadian rhythm, with morning levels being up to 40 % higher than afternoon levels [91, 92], although a peak during the afternoon has been observed by several authors [83, 93, 94]. Furthermore, the average concentration of cortisol in pigs decrease with age, reaching a stable profile around 20 weeks of age, when levels were about 37 % lower than at 12 weeks of age [91, 95]. In addition, gender is another source of variation, with concentration in barrows being about 15 % higher than in gilts [91].

Plasma or serum has been the most widely used samples to measure cortisol in pigs and they reflect both the fraction of protein-bound cortisol and the free cortisol [91, 96]. However, there is a growing tendency to use saliva for cortisol measurements because it can be obtained by non-invasive techniques and reflects only the free cortisol which is the active fraction [43, 83, 97].

Although most stress stimuli tend to increase cortisol, in some situations cortisol such levels remain unchanged and show a large variability between individuals. For example, after 24 and 48 h transport, cortisol concentrations were not modified [98], and similarly, cortisol concentrations were not affected by feed deprivation or isolation, with a large variability between individual pigs [25, 63]. Likewise, McGlone et al. [99] found no significant change in plasma cortisol levels after shipping.

### Hypothalamic-pituitary-gonadal axis: testosterone

Testosterone is a circulating androgen produced by the testes in males and by the ovaries in females, with a small amount being produced by the adrenal gland. The production of testosterone depends on the secretion of gonadotropin releasing hormone (GnRH) by the hypothalamus. GnRH binds to the gonadotrophs of the anterior pituitary to stimulate the secretion of luteinizing hormone (LH) and follicle stimulating hormone (FSH). FSH acts on Sertoli cells to aid spermatogenesis while LH induces the secretion of testosterone by Leydig cells in testes and by Theca cells in ovaries [100].

Although chronic stress has an inhibitory effect testosterone secretion [101], acute stress has been seen to increase testosterone levels in blood [102] and saliva [103]. For example, in pigs, an increase in salivary testosterone was detected after immobilization with a nose-snare or after short road transport [103]. The cause of this increase of testosterone in stress conditions is unclear, but could be due to increased sensitivity of the testes to LH, as a result of the activation of the SAM axis [102]. In addition, the administration of ACTH to boars induced a rapid increase in testosterone levels and adrenals could be a source of testosterone in stress situations [104].

### Immune System: acute phase proteins, immunoglobulin A and interleukin-18

#### Acute phase proteins

Acute phase proteins (APPs) are blood proteins whose concentration is modified in response to inflammation, infection, and physical or psychological stress [105]. They are primarily synthesized by the liver [106] and released into the systemic circulation to restore the homeostasis of the organism [107]. They serve as a tool for disease diagnosis in pigs, especially to detect acute inflammation, but they have also been studied as stress biomarkers in plasma [105, 108, 109] and saliva [110].

A hypothesis was developed by Murata [111] to explain the link between APP and stress. This hypothesis starts with the SAM and HPA axis activation. The release of catecholamines by the SAM leads to the induction of pro-inflammatory cytokines such as interleukin-6 [112], which is known to be a strong inducer of hepatic APP production in pigs [113]. In swine, numerous studies have measured APP in stressful conditions; however, there is no clear pattern of APP response in stress situations and in some cases contradictory results were obtained. For example, while haptoglobin levels increased after 20 min of transport and 3 h lairage [114], 45 min transport or social isolation produced no significant changes in this APP [110]. Another example is the case of serum amyloid A, which increased in isolated animals [110] but did not change after stress induced by changes in the pattern of food administration [115]. So, further studies would be needed to clarify to the link between stress and the acute phase response.

#### Immunoglobulin A

Immunoglobulin A (IgA) is the most abundant antibody in mucous membranes, where it has a protective role against infectious agents, allergens and foreign proteins [116]. IgA has been described as a stress biomarker in rats [117], dogs [118] and humans [119]. In pigs, an increase in saliva IgA has been reported after immobilization [84, 120], in endotoxemia [121], and after isolation [63]. In the case of isolation, IgA was more strongly affected than other markers such as cortisol and testosterone [63].

#### Interleukin-18

Interleukin-18 (IL-18*)* is a proinflammatory cytokine initially detected in Kupffer cells in mice, although it has been located in other sites such as adrenal cortex, astrocytes, microglia or keratinocytes [122, 123]. It is as an interferon-gamma inducing factor [122], and also has antitumor and antimicrobial activity [124, 125].

Based on its increase in rats after ACTH administration [126], IL-18 has been proposed as a stress biomarker. Although studies in domestic animals are very scarce, an increase in IL-18 concentration in saliva has been described in pigs after 1 h of immobilization [120].

## Conclusions

In this paper the main causes and consequences of stress in pigs, as well as the biomarkers that can be used for its evaluation are reviewed. Stress is a process with multifactorial causes, producing an organic response that generates negative effects in the health and production of the animals affected. Due to the diverse causes that can produce stress and the various physiological systems involved in the stress response, ideally a panel of various biomarkers should be used to assess the stress response in animals. We hope that this review can increase the understanding of the stress process, contribute to a better control and reduction of potential stressful stimuli in pigs, and encourage further studies and developments to better monitor, detect and manage stress on pig farms.

## Abbreviations

ACTH, adrenocorticotropic hormone; ADFI, average daily feed intake; ADG, average daily gain; APPs, acute phase proteins; BW, Body weight; CgA, chromogranin A; DFD, dark, firm, dry; FSH, follicle stimulating hormone; G:F, gain:feed ratio; GnRH, gonadotropin-releasing hormone; HPA, hypothalamic-pituitary-adrenal axis; HPG, hypothalamic-pituitary-gonadal axis; IgA, immunoglobulin A; IL-18, interleukin-18; LH, luteinizing hormone; PSE, pale, soft, exudative; SAM, Sympathetic-adrenal-medullary axis
